# Supplementary material for: Mutations in PCYT2 disrupt etherlipid biosynthesis and cause a complex hereditary spastic paraplegia
Source: Brain. 2019 Oct 22;142(11):3382–97. doi: 10.1093/brain/awz291 (PMC6821184; doi:10.1093/brain/awz291)
Supplement: awz291_Supplementary_Data [file awz291_supplementary_data.zip › awz291-Suppl_data/Supplementary_Data4.zip!/Supplementary information 4 - plasma lipidomics.zip!/Plasma lipidomics/Control vs Patient3b/Statistical plots and data/PC species/Boxplots all.pdf]

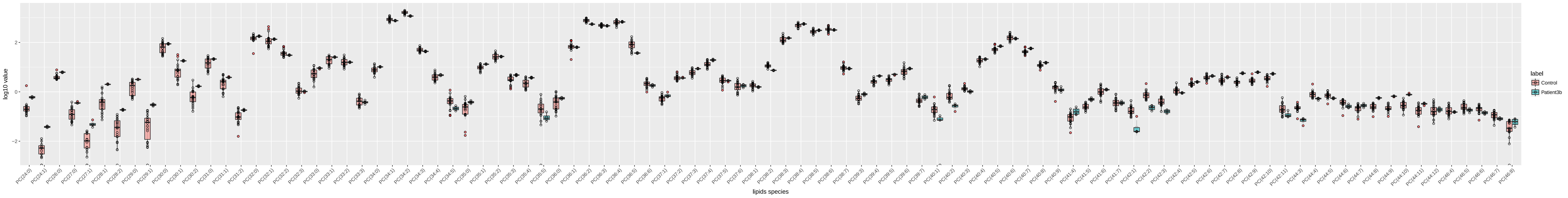
log10 value
2
0
−2
PC(24:0) PC(24:1) PC(26:0) PC(27:0) PC(27:1) PC(28:1) PC(28:2) PC(29:0) PC(29:1) PC(30:0) PC(30:1) PC(30:2) PC(31:0) PC(31:1) PC(31:2) PC(32:0) PC(32:1) PC(32:2) PC(32:3) PC(33:0) PC(33:1) PC(33:2) PC(33:3) PC(34:0) PC(34:1) PC(34:2) PC(34:3) PC(34:4) PC(34:5) PC(35:0) PC(35:1) PC(35:2) PC(35:3) PC(35:4) PC(35:5) PC(36:0) PC(36:1) PC(36:2) PC(36:3) PC(36:4) PC(36:5) PC(36:6) PC(37:1) PC(37:2) PC(37:3) PC(37:4) PC(37:5) PC(37:6) PC(38:1) PC(38:2) PC(38:3) PC(38:4) PC(38:5) PC(38:6) PC(38:7) PC(39:3) PC(39:4) PC(39:5) PC(39:6) PC(39:7) PC(40:1) PC(40:2) PC(40:3) PC(40:4) PC(40:5) PC(40:6) PC(40:7) PC(40:8) PC(40:9) PC(41:4) PC(41:5) PC(41:6) PC(41:7) PC(42:1) PC(42:2) PC(42:3) PC(42:4) PC(42:5) PC(42:6) PC(42:7) PC(42:8) PC(42:9) PC(42:10) PC(42:11) PC(44:3) PC(44:4) PC(44:5) PC(44:6) PC(44:7) PC(44:8) PC(44:9) PC(44:10) PC(44:11) PC(44:12) PC(46:4) PC(46:5) PC(46:6) PC(46:7) PC(46:8)
lipids species
label
Control
Patient3b
